# Effect of COVID-19 Lockdown in Spain on Structural and Functional Outcomes of Neovascular AMD Patients

**DOI:** 10.3390/jcm10163551

**Published:** 2021-08-12

**Authors:** Alicia Valverde-Megías, Daniela Rego-Lorca, José Ignacio Fernández-Vigo, Antonio Murciano-Cespedosa, Alicia Megías-Fresno, Julián García-Feijoo

**Affiliations:** 1 Department of Ophthalmology, San Carlos Clinical Hospital, 28040 Madrid, Spain; dpregolorca@gmail.com (D.R.-L.); jfvigo@hotmail.com (J.I.F.-V.); jgarciafeijoo@hotmail.com (J.G.-F.); 2 IdISSC (Instituto de Investigación Sanitaria del Hospital Clínico San Carlos), 28040 Madrid, Spain; 3 Centro Internacional de Oftalmología Avanzada, 28010 Madrid, Spain; 4 Department of Biomathematics, Faculty of Biology, Complutense University, 28040 Madrid, Spain; murciano@ucm.es; 5 Department of Biochemistry and Molecular Biology, Faculty of Biology, Complutense University, 28040 Madrid, Spain; amegias@bio.ucm.es; 6 Department of Opthalmology, Faculty of Medicine, Complutense University, 28040 Madrid, Spain

**Keywords:** COVID-19, nAMD, anti-VEGF

## Abstract

This is a retrospective single-center study of patients with neovascular age-related macular degeneration whose follow-up was delayed due to COVID-19 pandemic with at least three months between visits in Madrid, Spain. The purpose of the study was to evaluate best corrected visual acuity (BCVA) changes and try to identify features in optical coherence tomography (OCT) that could be related to more profound visual loss. It included 270 eyes. The two last visits before lockdown were used for comparison with the visit after lockdown. BCVA changed from 60.2 ± 18.2 to 55.9 ± 20.5 ETDRS letters. 29% of the eyes lost more than 5 letters. OCT was active in 67% of eyes before lockdown and in 80.4% after lockdown. Multiple lineal analysis showed that patients whose OCT before lockdown presented with a combination of intra and subretinal fluid were more likely to suffer a greater visual loss (*p* = 0.002). These patients should be encouraged to not miss any visits in case a new lockdown is imposed.

## 1. Introduction

Starting in December 2019 in China [1], severe acute respiratory syndrome coronavirus type 2 (SARS-CoV-2) spread all over the world, reaching Spain with the first confirmed case on the 31 January 2020. A very strict national lockdown was imposed on the 14 March until its lifting the 21 June. With a total of 124,880 reported confirmed cases (and 28,324 reported deaths) by the end of the lockdown [2], Spain was the third most affected country in Europe after Russia and United Kingdom. Among Spanish territory, community of Madrid held the highest incidence and mortality rate [2]. Neovascular age related macular degeneration (nAMD) in Spain mainly affects patients over the age of 70. This very group is the one showing a higher COVID mortality rate [3]. Consequently, fear of contact with COVID-19 patients and difficult access to hospital as main factors dramatically reduced follow-up visits and treatment compliance of our nAMD patients, not only during the three months of lockdown, but also expanding for several months thereafter. Functional and anatomical success in the treatment for nAMD is subdued to the ability to maintain the macula free of exudation from choroidal neovascularization with anti-VEGF injections while minimizing the development of atrophy and fibrosis [4]. Different protocols have been developed in order to adjust number of visits, number of injections and vision changes with intervals never longer than 12 weeks [5,6]. The AURA study, a multi-country real-life experience of anti-VEGF therapy for nAMD, demonstrated that less frequent visits and injections were associated with limited improvements in visual outcome [7]. The COVID-19 pandemic prompted unprecedented delays to treatment for patients receiving anti-VEGF intravitreal injections and unfortunately provided us with the unique opportunity to study the consequences of temporary treatment suspension on these patients.

We hereby describe our experience as a public major Tertiary Ophthalmology referral hospital in Madrid. The purpose of our study was to provide real world data on the impact of delayed treatment due to COVID-19 lockdown on structural and functional outcomes of neovascular AMD patients and try to identify those patients at greatest risk of visual loss due to delayed review. This information may be relevant if prioritisation of therapy is necessary in future emergency settings

## 2. Materials and Methods

### 2.1. Study Participants

In this consecutive observational case series, all patients from San Carlos Clinical Hospital (Madrid, Spain) under anti-VEGF treatment for exudative AMD the year prior to the lockdown were followed. The study adhered to the 1964 Helsinki declaration and its later amendments and was approved by San Carlos Clinical Hospital Ethics Committee. Written informed consent to use their medical information in the study analysis was routinely provided by all of the patients.

**Figure 1:**
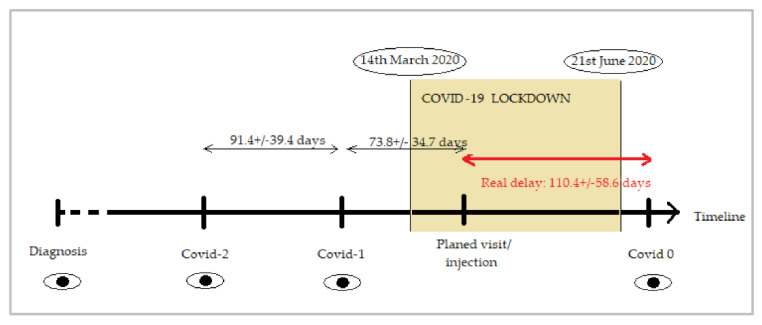
Graph showing the timeline and visits included in the study. The period of COVID-19 lockdown lasted from 14 March 2020 to 21 June 2020. *Covid-2* and *Covid-1* are the two last visits before the restriction period where the visit/injection was scheduled. *Covid 0* corresponds to the first visit after the lockdown period ends. Real delay (in red) was calculated as time from their planned visit/injection to the date the patients resumed their follow-up at *Covid 0* visit.

Inclusion criteria for this study were: (i) the resuming of follow-up after the 14th of March and, (ii) a period of at least twelve weeks between the visits before and after lockdown onset to prevent confounding factors with more usual delays in clinical practice. Furthermore, patients were required to have records of complete ophthalmological examinations carried out during the immediate two visits before lockdown, (named *Covid-1* and *Covid-2*) and the visit after the lockdown onset (*Covid 0*) (Figure 1).

Exclusion criteria were: (i) choroidal neovascularization (CNV) due to causes other than AMD, (ii) patients returning to hospital for visit *Covid 0* after data included in the present study were collected (January 2021), (iii) visual acuity of counting fingers or less before lockdown and, (iv) loading dose not completed before lockdown.

At all visits patients received a complete ophthalmological examination including: measurement of best corrected visual acuity (BCVA) using an *Early Treatment Diabetic Retinopathy Study* (ETDRS) letters chart, dilated ophthalmoscopy, slit lamp biomicroscopy, dilated fundus examination, structural optical coherence tomography (OCT) imaging and, when prescribed, anti-VEGF intravitreal injection. For each patient information about diagnosis (date, type of neovascularization and baseline BCVA), number of intravitreal injections one and two years before lockdown, anti-VEGF used and protocol applied was extracted from medical records. For the analysis, and to account for the variability inherent to this disease, we used data from the immediate two visits before lockdown (*Covid-2* and *Covid-1*) and the visit after the onset of the lockdown (*Covid 0*).

### 2.2. OCT Imaging 

Structural OCT imaging was performed with the Heidelberg Spectralis OCT device (Heidelberg Engineering, Heidelberg, Germany). Each set of scans included 25 horizontal B-scans, centered on the fovea, with a minimum strength signal of 25 as recommended [8]. Exudative disease activity was assessed as active/inactive CNV and presence of subretinal fluid (SRF) and intraretinal fluid (IRF). Macular cystoid edema was recorded and central retinal thickness (CRT) was also measured. Structural OCT images were reviewed by two independent and experienced readers (A.V.M. and D.R.L.). 

### 2.3. Statistical Analysis 

Statistical calculations were performed using STATA (StataCorp, version 15). 

Normality of variables was assessed with Mann Whitney test. Statistical significance of the differences for binomial variables was assessed using proportion comparisons with normal approximation. Generalized linear models (GLMs) were used to assess the relationship between changes in BCVA (dependent variable) and other clinical features and demographics (independent variables). *Post hoc* analyses were performed with Wald tests. A *p* value < 0.05 was considered statistically significant.

## 3. Results

### 3.1. Characteristics of the Eyes Included in the Analysis 

**Figure 2:**
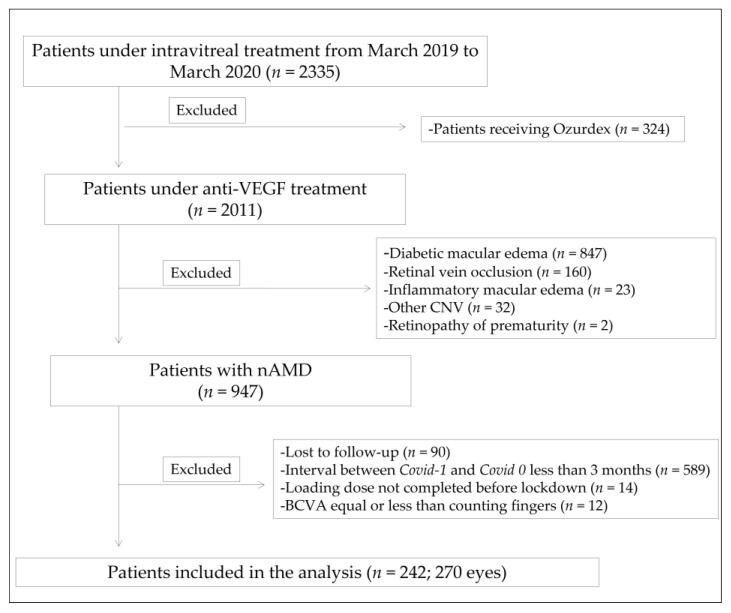
Flowchart of the study. Anti-VEGF: anti-vascular endothelial growth factor; CNV: choroidal neovascularization; nAMD: neovascular age related macular degeneration; BCVA: best corrected visual acuity.

The year before the lockdown (from 13 March 2019 and 2020), 2335 patients were under intravitreal treatment at San Carlos Clinical Hospital, in Madrid. Of these, 324 were receiving dexamethasone implant (Ozurdex) and the remaining 2011 were receiving anti-VEGF agents. Discarding diagnoses such as diabetic macular edema, retinal vein occlusion and CNV with etiology different from AMD, 947 patients diagnosed with neovascular AMD were selected. Further application of exclusion criteria resulted in the final data set for this study: 270 eyes of 242 patients (flow chart available in Figure 2).

The baseline demographic data of eyes included in the analysis are shown in Table 1. The year of diagnosis of exudative AMD ranged from 2006 to 2020. All types of CNV were represented, although the most common was type 1 (66%) and the least common (1.8%), what we used to call polypoidal choroidal vasculopathy (nowadays formally known as aneurysmal type 1 neovascularization). Anti-VEGF agent used included ranibizumab (38.5%), aflibercept (36.7%) and bevacizumab (24.8%). The number of injections in the year preceding the lockdown was 5.38 ± 1.82 (range 1–11). Real delay (110.4 ± 58.6 days) was calculated as time from their planned visit to the date the patients resumed their follow-up as indicated in Figure 1. 

### 3.2. Functional and Anatomic Outcomes

Table 2 shows BCVA at time of diagnosis, and in the visits *Covid-2, Covid-1* and *Covid 0*. Mean *Covid-1* BCVA was 60.2 ETDRS letters. No significant differences were found between BCVA in *Covid-2* and *Covid-1* visits. BCVA in *Covid 0* visit was 55.9 letters. Therefore, a total mean number of 4.23 ETDRS letters were lost when the follow-up was resumed (*p* < 0.001). 

**Figure 3:**
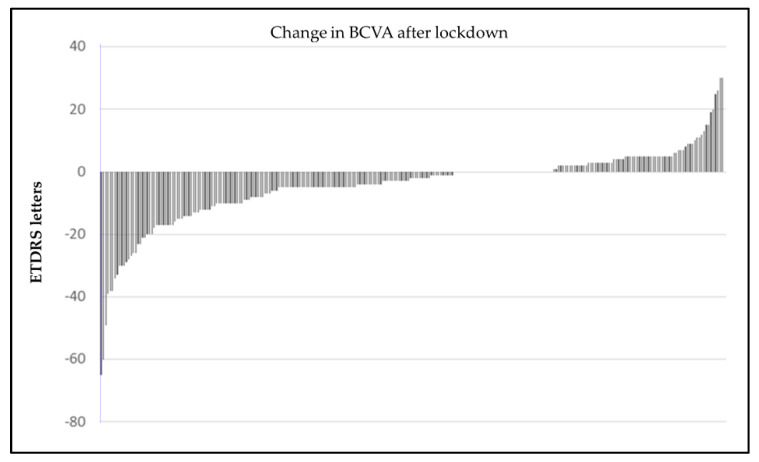
Difference in BCVA between *Covid-1* and *Covid 0*. Each bar represents one eye. Negative values are letters lost after lockdown. Positive values are letters gained.

**Figure 4:**
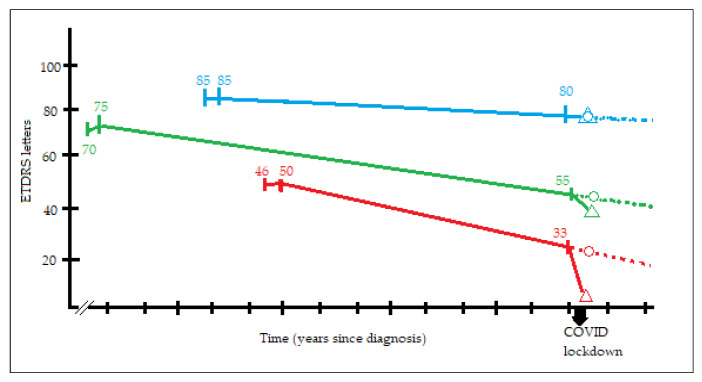
Graph showing three representative examples (blue, green and red lines) of visual decay approximation calculated for each patient of our study. In every line there are three points with numbers (visual acuity in ETDRS letters) and two symbols. The first point is visual acuity at diagnosis at the corresponding year. The second point is visual acuity after loading dose. The third point is visual acuity at *Covid-1*. From the second point to the third, we have considered a line, that has been prolonged (dotted line) matching expected visual acuity at *Covid 0* (represented as a circle). Actual visual acuity at *Covid 0* is represented as a triangle. For each patient in our study, the difference in *Covid 0* between expected visual acuity and actual visual acuity is the visual loss we have attributed to COVID-19 lockdown mediated delays in follow-up and intravitreal injections.

Distribution of individual changes in visual acuity is shown in Figure 3. In this unusual situation of prolonged visit intervals, visual acuity decay even in properly treated nAMD must be accounted for. For each patient, we used medical records to calculate the rate of ETDRS letters lost per year, starting from visual acuity after loading dose in the year of diagnosis, and spanning to BCVA in *Covid-1*. Then, we applied the individual rate of visual acuity loss to the time each patient was absent from follow-up. The resulting number of letters was considered as loss not attributable to delay in follow-up, but merely to “natural history of treated nAMD” (Figure 4). In our cohort, the mean loss of 4.23 ETDRS letters was distributed as follows: a mean of 0.6 letters associated with unavoidable decrease and a mean of 3.63 letters attributable to COVID-19 lockdown itself (paired sign rank test, *p* < 0.001).

**Figure 5:**
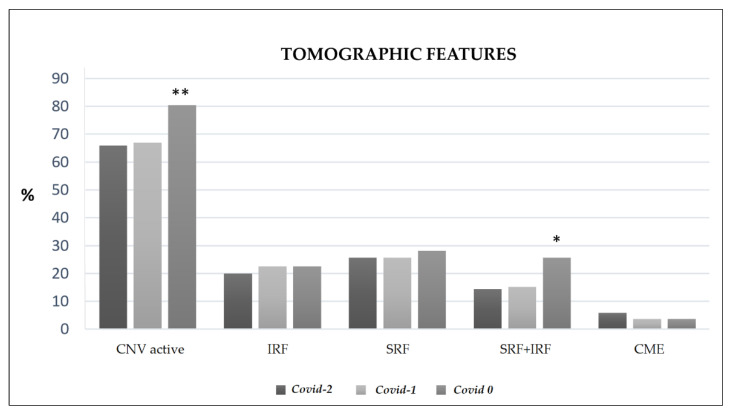
Percentage of eyes with different tomographic features. CNV: choroidal neovascularization; IRF: intrarretinal fluid; SRF: subretinal fluid; CME: cystoid macular edema. ** *p* < 0.001, * *p* < 0.01, for *Covid 0* vs. *Covid-1* comparison.

Structural parameters and tomographic features determined by OCT imaging are shown in Table 3 and Figure 5. OCT evaluation revealed CNV active in 65.9% of eyes in *Covid-2*, 67% in *Covid-1*, and 80.4% in *Covid 0* visits. Wilcoxon signed rank test for comparison of the proportion of active CNV showed no significant differences between *Covid-2* and *Covid-1* values (*p* = 0.78) whereas statistical significance was found when comparing *Covid-1* with *Covid 0* (*p* = 0.0004). CRT remained stable in the last two visits before lockdown but increased significantly in *Covid 0* compared to *Covid-1* visit (304.4 vs. 347.5 microns, *p* < 0.001). Regarding the distribution of fluid in the different tomographic compartments, COVID-19 lockdown increased the proportion of patients with coexisting intra and subretinal fluid (15.2% in *Covid-1* vs. 25.6% in *Covid 0, p* < 0.01).

These results show a general worsening of both functional and tomographic features as a consequence of treatment discontinuation, with differential impact depending on OCT phenotype before lockdown, considering in this context phenotype exclusively as fluid distribution.

### 3.3. Further Analysis of BCVA Changes

In general terms, the changes in BCVA can be subdivided into three groups: (i) those patients in which BCVA after lockdown was within ± 5 ETDRS letters compared to BCVA before lockdown, (ii) patients losing more than 5 EDTRS letters, (iii) patients gaining more than 5 ETDRS letters. The first group comprised 63% of the eyes, the second 29% and the third 8%. Although there were no differences in baseline characteristics among these groups, eyes improving visual acuity tended to have low visual acuity and fibrotic central CNV before COVID-19.

The results of the study of the association between change in BCVA and other variables (age, months from diagnosis, CNV type, BCVA at diagnosis, *Covid-2,* and *Covid-1*, anti-VEGF used, treatment regimen, number of injections, delay in visits and location of fluid in OCT before and after COVID) are shown in Table 4. BCVA in *Covid-1*, representative of functional status before lockdown, showed a trend towards significance *(p* = 0.06). This suggests that patients with better visual acuity were more likely to lose more ETDRS letters.

CNV activity in OCT in *Covid-1* was directly related to a greater visual acuity loss. OCT phenotype before lockdown was associated with BCVA loss as follows: considering the “absence of fluid” as reference, presence of sub and intraretinal fluid combined carried a risk of losing 6.8 EDTRS letters (*p* = 0.002), presence of subretinal fluid alone was related to a loss of 3.8 letters (*p* = 0.039), cystoid macular edema a loss of 2.6 letters and intraretinal fluid alone a loss of 1.7 letters (*p* non-significant). When comparing among active phenotypes, eyes with combination of sub and intraretinal fluid had a visual loss 5 letters greater than eyes with intraretinal fluid alone (*p* = 0.025).

The type of CNV, type of anti-VEGF used, regimen employed and days of delay were not related to functional outcome.

## 4. Discussion

COVID-19 lockdown has imposed worldwide great delays in the treatment of nAMD patients for the first time in anti-VEGF era and its negative impact has been unevenly distributed among our nAMD patients.

MARINA [9] and ANCHOR [10] clinical trials showed us that anti-VEGF agents administered in a monthly basis improved BCVA in nAMD and achieved functional stabilization. PIER study [11] taught us that fixed quarterly administration, however, resulted in visual loss. A somehow premonitory study [12] published in 2020 showed the effects of delayed retreatment in nAMD. And then, the pandemic started.

General guidelines on the management of nAMD were prepared after the onset of the pandemic [13,14], recommending treatment administration for every nAMD patient every two months, regardless visual functional or macular anatomical aspects, in an empirical attempt to minimize what was expected to be a mayor disaster. It is clear that this catastrophic situation has had a negative impact on functional and anatomic outcome of nAMD, as can be seen in our cohort and also in the majority the studies published in 2021 about this subject [15,16,17,18,19,20,21,22]. Even with an average delay of one month, Borrelli et al. [15] found significant loss of BCVA and proportional to visit delay. Our study was focused on patients missing appointments for more than three months, and although we found a mean loss of 3.63 ETDRS letters, it was not related to time of delay and 71% of the eyes retained reasonably good visual acuity.

There are some noticeable differences in the study populations between our study and that by Borrelli. First, interval between visits before lockdown in our study tended to be shorter. Second, bevacizumab was the predominant drug used and in a *pro re nata* approach, while we used bevacizumab, aflibercept and ranibizumab alike and different regimes were represented (treat and extend-TAE, fixed and *pro re nata*). We did have, nevertheless, some similarities such as the percentage of patients with active OCT before COVID-19 (above 60%).

In the study by Naravane et al. [16], 36 patients diagnosed with neovascular AMD experienced delays in treatment (defined as more than 14 days) and visual acuity decreased 6.2 letters (from 48.6 to 42.3, *p* = 0.04). In our study with 270 eyes, mean delay of 110 days meant a change of −3.6 letters. Disparity of results could be related to different baseline BCVA in both cohorts (48.6 vs. 60.2) and different criteria applied for delay definition. On the other hand, percentage of active OCT was not provided in their study.

In the study by Sevik et al. [20], a delay of three months (13.9 ± 6.2 weeks) considered in 33 patients, under a TAE regimen with aflibercept, with mean BCVA of 59 ETDRS letters and only 36.4% of eyes with active OCT at baseline had more severe consequences than those found in our study: 50 ETDRS letters and 60.6% of eyes with active OCT after COVID-19 lockdown. The mean age of turkish nAMD patients is more than 13 years younger than our cohort, so maybe different genetics or other factors could be playing a role.

The effect of lockdown in United Kingdom was reported by Stone [21], in an elegant study including nAMD, retinal vein occlusion and diabetic macular edema patients. In the sub-analysis for nAMD patients, mainly under TAE regimen, 61.9% of the delayed group, defined as 8 or more weeks delay in their appointment (*n* = 194) maintained vision (lost less than 5 letters). Visual acuity before and after lockdown were similar to ours (60.1 vs. 60.2, and 55.2 vs. 55.9, respectively). OCT was still inactive in 27% of the delayed group at the follow-up visit after lockdown (20% in our study). About the distribution of fluid in OCT, they do not show data on OCT before COVID-19, so no prediction of high risk patients is offered, although the proportion of eyes after COVID-19 with combination of intra and subretinal fluid is 12% in eyes maintaining vision and 35% in eyes losing more than 5 letters. Also, in our data, OCT after COVID-19 with combination of intra and subretinal fluid was related to a greater visual loss.

It is essential to ascertain how many of those ETDRS letters that were lost during the restrictions of the pandemic will be recoverable with adequate treatment afterwards. We grouped the eyes losing more than 5 letters (29% in our sample) using this provisional threshold based on findings in the PIER study [11] as a possible point-of-no-return after lockdown. In PIER, a clinical trial, after the loading dose, injections were administered every three months. By month 24, a change of −2.2 and −2.3 letters was found for the dose of 0.3 and 0.5 mg of ranibizumab, respectively. In the second amendment of the protocol, 87 patients were rolled over from quarterly to monthly injections. The result was a gain of 2.2 and 4.1 letters in the 0.3 mg and 0.5 mg after rollover, respectively. If these findings could be applied to COVID-19 lockdown, it would lead us into expecting some improvement in patients losing less than 5 letters over time with reestablishment of proper treatment. However, real life studies find that after six months of proper treatment, although percentage of active OCT is restored, BCVA is not [19].

The new variants of SARS-CoV-2 coronavirus might lead to new confinements in the future and we must dedicate all our efforts especially to patients in which delay in treatments would lead to severe visual loss. If we use the loss of more than 5 ETDRS letters as a valid threshold, this study shows that OCT phenotype can help identify the patients at a higher risk in case of nAMD treatment suspension: those patients with active CNV in OCT with a combination of intra and subretinal fluid were prone to losing a mean of 6.8 letters. Thus, in case of a new lockdown or severe restrictions, reducing the number of patients to a minimum (15% of our cohort had this phenotype) would enable us to provide a safer environment with better distancing among patients. Also, we could provide these patients with well-founded information about the visual consequences of staying home. In the reverse in patients with less susceptible OCT phenotypes, not absolutely necessary visits to hospital (carrying unjustified life-threatening risk due to Covid) could be avoided.

This study reflects the situation of a single center and might not be applicable to other centers in our country. For instance, in a recent study by Arruabarrena et al. [22], the cohort from University Hospital of Alcalá de Henares, a subset of 144 patients, also in the community of Madrid, had substantially lower BCVA before lockdown (56.8 letters, similar to our BCVA after lockdown in our heavily delayed cohort). However, the percentage of inactive OCT at baseline was 44%.

Other limitations of this study are possible bias associated: as in any real clinical practice scenario, anti-VEGF agents used are not randomly assigned (bevacizumab is the drug normally used in our service in fixed regimes and almost never in TAE regimes). Also, a patient perceiving visual loss might be more motivated not to miss a programmed visit. In addition, it should be noted that a significant number of our nAMD patients were excluded from the study due to strict criteria applied for inclusion and this has the potential to affect generalizability of our findings.

Strengths of this study are the high number of eyes included with completely documented follow-up before and after COVID-19, the strict inclusion criteria in terms of real delay, the concept of natural history visual loss in longer than usual appointment intervals, and diversity of regimes and anti-VEGF drugs used.

## 5. Conclusions

In conclusion, prolonged delayed attention in nAMD due to COVID-19 restrictions resulted in general visual deterioration of 3.6 letters in our Hospital. A reasonable percentage of delayed patients maintained their prior visual acuity. Eyes with CNV active in pre-lockdown OCT assessments displaying combination of intra and subretinal fluid were the most severely affected.

## Figures and Tables

**Table 1:** Baseline demographic data of eyes included in the study.

Age (years), mean (SD)	82.8 (6.5)
Patients with both eyes eligible (%)	28 (11.6)
Years since CNV diagnosis, mean (SD; range)	4 (3; 0.14–13.4)
CNV type, *n* (%)	
Type 1	178 (66%)
Type 2	56 (20.7%)
Type 3	31 (11.5%)
AT-1	5 (1.8%)
Anti-VEGF used, *n* (%)	
Ranibizumab	104 (38.5%)
Aflibercept	99 (36.7%)
Bevacizumab	67 (24.8%)
Regimen	
*Pro re nata*	115 (42.6%)
Treat-and-extend	40 (14.8%)
Fixed	115 (42.6%)
Anti-VEGF injections, mean (SD; range)	5.38 (1.82; 1–11)
Delay in follow-up/treatment (days), mean (SD; range)	110 (56.3; 28–340)

SD standard deviation, *n* number of eyes, CNV choroidal neovascularization, AT-1 aneurysmal type 1 neovascularization, VEGF vascular endothelial growth factor.

**Table 2:** Functional parameters at different times of the study.

	ETDRS Letters, Mean (SD, SE; Range)	*p* Value *
Diagnosis BCVA	62.6 (16.9, 1.0; 5–90)	0.02
Covid-2 BCVA	60.7 (18.4, 1.1; 5–91)	0.3
Covid-1 BCVA	60.2 (18.2, 1.1; 5–85)	
Covid 0 BCVA	55.9 (20.5, 1.2; 5–90)	<0.001

ETDRS Early treatment diabetic retinopathy study, BCVA best corrected visual acuity, SD standard deviation, *SE* standard error. * *Covid-1* BCVA used for comparisons.

**Table 3:** Structural parameters at different times of the study.

	***Covid-2***	***Covid-1***	***Covid 0***
CRT measured with OCT, mean (SD; range)	310.3 (165.6; 83–914)	304.4 (162; 50–1088)	347.5 (188.9; 100–1261)
*p* value *	0.32		**<0.001**
OCT with active CNV, *n* (%)	178 (65.9)	181 (67)	217 (80.4)
*p* value *	0.78		**<0.001**
Intraretinal fluid, *n* (%)	54 (20)	61 (22.6)	61 (22.6)
*p* value *	0.46		1
Subretinal fluid, *n* (%)	69 (25.6)	69 (25.6)	76 (28.1)
*p* value *	1		0.51
Both intra and subretinal fluid, *n* (%)	39 (14.4)	41 (15.2)	69 (25.6)
*p* value *	0.79		**<0.01**
Cystoid macular edema, *n* (%)	16 (5.9)	10 (3.7)	10 (3.7)
*p* value *	0.23		0.81

CRT central retinal thickness, OCT optical coherence tomography, SD standard deviation, CNV choroidal neovascularization, *n* number of eyes. * *p* value for comparison with *Covid-1* visit.

**Table 4:** Effect of the variables in the study and the functional outcome.

Variable	*p* Value ^1^	Variable	*p* Value ^1^
Age	0.107	**Active CNV in OCT in *Covid-1***	0.028
Months since diagnosis	0.639	**Subretinal fluid**	0.039 *
CNV type	0.992	Intrarretinal fluid	0.36 *
BCVA at diagnosis	0.815	**Sub and intrarretinal fluid**	0.002 *
*Covid-2* BCVA	0.530	Cystoid macular edema	0.50 *
*Covid-1* BCVA	0.060		
Anti-VEGF used	0.622	**Active CNV in OCT in *Covid 0***	<0.0001
Treatment regimen	0.061	**Subretinal fluid**	0.001 *
Number of injections the year before ^2^	0.207	Intrarretinal fluid	0.21 *
Days since last visit	0.351	**Sub and intrarretinal fluid**	<0.0001 *
Days from scheduled visit	0.509	Cystoid macular edema	0.19 *

CNV choroidal neovascularization, VEGF vascular endothelial growth factor; ^1^ Generalized linear models used for qualitative variables and simple lineal regression for quantitative variables; ^2^ Less than four injections vs. four or more; * *p* values for comparisons with OCT without fluid.

## Data Availability

The data used to support the findings presented in this study are available on request from the corresponding author.
